# Identification of a novel nonsense mutation in *SH2D1A* in a patient with X-linked lymphoproliferative syndrome type 1: a case report

**DOI:** 10.1186/s12881-018-0576-y

**Published:** 2018-04-12

**Authors:** Xiaodong Lyu, Zhen Guo, Yangwei Li, Ruihua Fan, Yongping Song

**Affiliations:** 1 Central Laboratory, the Affiliated Cancer Hospital of Zhengzhou University, Henan Cancer Hospital, Zhengzhou, 450000 Henan China; 2 Department of Hematology, the Affiliated Cancer Hospital of Zhengzhou University, Henan Cancer Hospital, Zhengzhou, 450000 Henan China

**Keywords:** XLP1, *SH2D1A*, Nonsense mutation, Amplicon sequencing

## Abstract

**Background:**

X-linked lymphoproliferative syndrome type 1 (XLP1) is an X-linked recessive genetic disorder with a strong resemblance to hemophagocytic lymphohistiocytosis (HLH). Causative mutations for XLP1 have been identified in *SH2D1A*, located on chromosome Xq25.

**Case presentation:**

We report a case of an 18-month-old male with a novel nonsense mutation in *SH2D1A*. The patient presented the typical phenotype of HLH, including splenomegaly and hemophagocytosis in the bone marrow. Thus, he was initially diagnosed with HLH based on HLH-2004 guidelines. High-throughput amplicon sequencing was performed to detect mutations in the most commonly reported causative genes of HLH, i.e., *PRF1, UNC13D, STX11, STXBP2, SH2D1A,* and *XIAP.* A likely pathogenic nonsense mutation was detected in *SH2D1A* (NM_002351.4:c.300T>A). The mutation was inherited from the patient’s mother, and an X-linked recessive mode of inheritance was confirmed by a two-generation pedigree analysis based on Sanger sequencing results.

**Conclusions:**

The nonsense mutation in *SH2D1A* (NM_002351.4:c.300T>A) was reported for the first time in a case of XLP1 and was considered to be likely pathogenic based on the truncation of the mRNA sequence. This finding expands the spectrum of known XLP-related mutations in Chinese patients and indicates the utility of amplicon sequencing for XLP and HLH diagnosis.

**Electronic supplementary material:**

The online version of this article (10.1186/s12881-018-0576-y) contains supplementary material, which is available to authorized users.

## Background

X-linked lymphoproliferative syndrome type 1 (XLP1; OMIM 308240), also known as Duncan disease, was initially reported in the 1970s [1]. It is an X-linked recessive genetic disorder caused by mutations in the *SH2D1A* gene, which is located on chromosome Xq25. *SH2D1A* encodes a src-homology 2 (SH2) domain in a signal-transducing protein, i.e., SLAM-associated protein (SAP), which is important for signaling events activating T and NK cells [2]. The phenotype of XLP1 has a strong resemblance to that of hemophagocytic lymphohistiocytosis (HLH) [3], including fever, splenomegaly, and cytopenia. XLP1 is often triggered by Epstein–Barr virus infection [4]. The incidence of XLP in the United States was estimated to be approximately 1 in 1 million males [5], but the incidence and mutation spectrum of XLP in China remain unknown owing to limited epidemiological data.

We report an 18-month-old male patient who was initially diagnosed with HLH based on clinical examination. However, a diagnosis of XLP1 was confirmed based on amplicon sequencing results, and a novel pathogenic nonsense mutation (c.300T>A) in *SH2D1A* was identified. Furthermore, a two-generation pedigree analysis was performed to confirm the mode of inheritance of the mutation in the patient.

## Case presentation

The study was approved by the institutional ethics committee of the Affiliated Cancer Hospital of Zhengzhou University. The CARE guidelines were followed in reporting this case. Written informed consent was obtained from the patient’s parents before collecting blood samples.

**Fig. 1:**
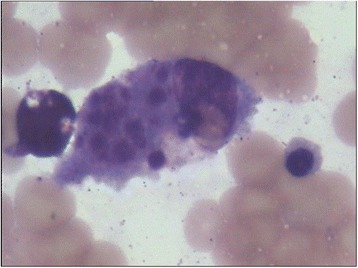
Bone marrow examination. Phagocytosis was clearly observed in the bone marrow, but no evidence of malignancy was observed

The patient was an 18-month-old male of Han Chinese ethnicity (with family from Henan province, China) who had intermittent fever for 2 weeks and was admitted to the Affiliated Cancer Hospital of Zhengzhou University. His temperature was 39.0 °C, and hepatomegaly was observed during an ultrasound examination. Blood analysis revealed elevated C-reactive protein levels, mild pancytopenia, low NK cell activity (4.65%), and low plasma albumin levels (18.8 g/L). Hemophagocytosis with no evidence of malignancy was observed during bone marrow examination (Fig. 1). The laboratory test results suggested the typical phenotype of HLH, based on HLH-2004 guidelines [6].

Genomic DNA was extracted from peripheral blood mononuclear cells (PBMCs) using the QIAamp Blood Kit (Qiagen, Hilden, Germany) according to the manufacturer’s protocol. Multiple PCR primers were designed using the Ion AmpliSeq™ Designer (https://www.ampliseq.com) to amplify the full-length exons of the 6 most common causative genes of HLH (*PRF1, UNC13D, STX11, STXBP2, SH2D1A,* and *XIAP*). PCR products were processed and sequenced using the S5XL genetic analyzer (Applied Biosystems®, Life Technologies, Grand Island, NY, USA), and mutations were identified using VariantCaller V1.0.

Eleven mutations were detected, including 8 heterozygous and 3 homozygous mutations (Table 1). All mutations were identified as polymorphisms based on sequences in current databases and clinical reports, except for the mutation NM_002351.4:c.300T>A (p.Tyr100X), which has not been reported to date. This mutation in exon 3 of *SH2D1A*, located on chromosome Xq25 (NC_000023.10:g.123504124, GRCh37), converts the codon for tyrosine to a stop codon (TAT>TAA). Several previously reported mutations in *SH2D1A* are involved in the pathogenesis of XLP1 and could lead to a similar phenotype to that of HLH [7, 8].

**Table 1:** Identified mutations in the patient with XLP1

Gene-exon	Position	Type	Zygosity	Frequency of variant (%)	ID in dbSNP	^a^ Allele frequencies in 1000 Genomes
PRF1-Exon3	c.900C>T	Synonymous	Heterozygous	50	rs885822	0.6959
PRF1-Exon3	c.822C>T	Synonymous	Heterozygous	49	rs885821	0.1126
UNC13D-Exon32	c.3198A>G	Synonymous	Heterozygous	54	rs7210574	0.5262
UNC13D-Exon27	c.2599A>G	Missense	Heterozygous	46	rs1135688	0.4994
UNC13D-Exon21	c.1992+5G>A	Intron mutation	Heterozygous	55	rs17581728	0.1575
UNC13D-Exon11	c.888G>C	Synonymous	Heterozygous	51	rs7223416	0.5098
STXBP2-Exon10	c.849G>A	Synonymous	Heterozygous	47	rs34450592	0.0124
STXBP2-Exon18	c.1576A>G	Missense	Homozygous	100	rs6791	0.7061
STXBP2-Exon18	c.1663A>G	Missense	Heterozygous	65	rs61736586	0.0122
XIAP-Exon7	c.^a^12A>G	Non-coding region	Hemizygote	100	rs28382740	0.2673
SH2D1A-Exon3	c.300T>A	Nonsense	Hemizygote	100	not applicable	not applicable

^a^ Allele frequencies were collected from The 1000 Genome Project: phase 3 (https://www.ncbi.nlm.nih.gov/variation/tools/1000genomes)

**Fig. 2:**
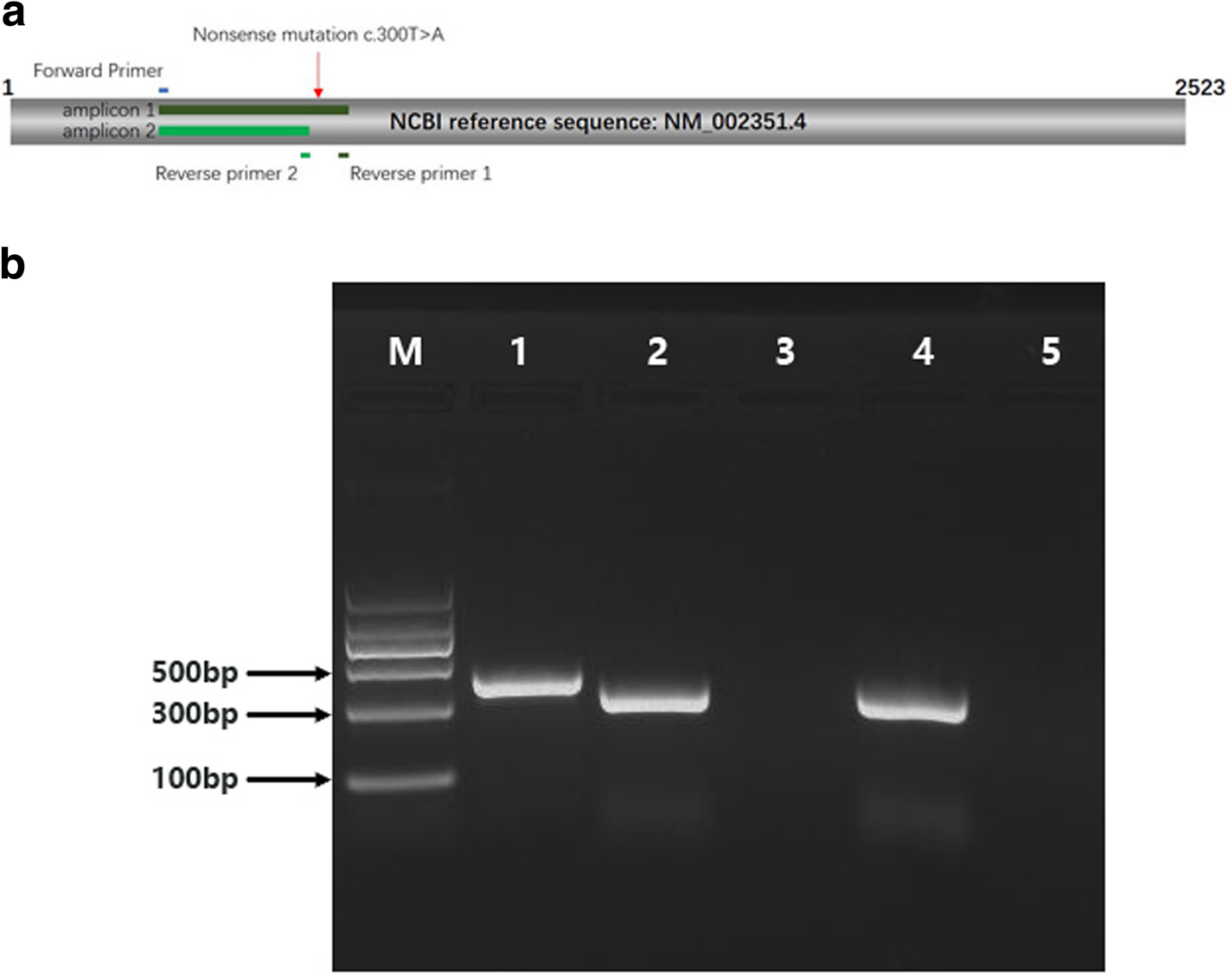
RT-PCR analysis of the *SH2D1A* gene. **a** Primer pairs and amplicons in the cDNA sequence of the *SH2D1A* gene. **b** Gel electrophoresis of RT-PCR products for the *SH2D1A* gene. M: DNA marker; lane 1: amplicon 1 of the control sample; lane 2: amplicon 2 of the control sample; lane 3: amplicon 1 of the patient’s sample; lane 4: amplicon 2 of the patient’s sample; lane 5: negative control

To investigate the impact of this mutation on mRNA transcription, we performed RT-PCR with total RNA extracted from the blood sample obtained from the patient. Then, cDNA was synthesized using SuperScript IV Reverse Transcriptase (Thermo Fisher Scientific, Waltham, MA, USA) according to the manufacturer’s protocol. PCR primers were designed using primer-BLAST (https://www.ncbi.nlm.nih.gov/tools/primer-blast) (Fig. 2a and Additional file 1: Table S1) to confirm the truncated cDNA sequence caused by the nonsense mutation. A full-length cDNA clone of the *SH2D1A* gene (SC118690; OriGene Technologies, Beijing, China) was included as control sample. For the full-length cDNA clone, PCR products were obtained using both pairs of primers, resulting in DNA fragments of 409 and 324 bp, respectively (Fig. 2b, lane 1 and lane 2). For the truncated cDNA from the patient, amplification was only possible using the second pair of primers due to the mutation (Fig. 2b, lane 3 and lane 4). Gel electrophoresis showed that the mRNA sequence of the *SH2D1A* gene in the patient was truncated, suggesting a loss or partial loss of protein function.

**Fig. 3:**
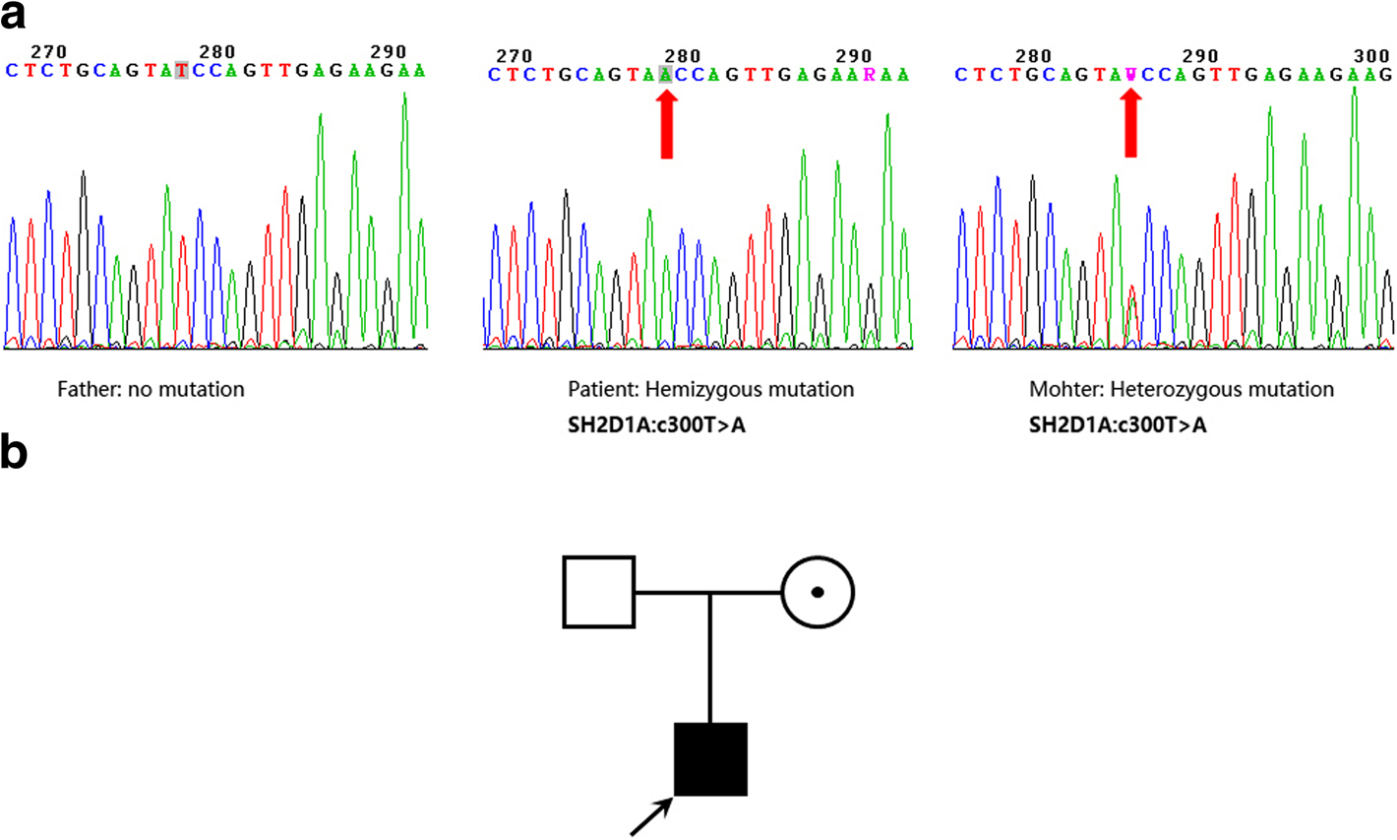
Two-generation pedigree analysis. **a** Sanger sequencing results of the amplified fragment in *SH2D1A* exon 3. The red arrows indicate the position of the identified mutation. **b** Family pedigree of the *SH2D1A* mutation found in the patient. The white square represents the father who is normal in this case, the white circle with a dot represents the mother who is a carrier of the X-linked recessive genetic disorder, the black square with an arrow represents the patient who is the proband

To confirm the mutation source, we performed a two-generation pedigree analysis using Sanger sequencing. The Sanger sequencing results were identical to those obtained by amplicon sequencing. The nonsense mutation in *SH2D1A* was carried by the patient’s mother, who showed a typical heterozygous profile (Fig. 3). This result confirmed an X-linked recessive inheritance pattern.

## Discussion and conclusions

We present the case of an 18-month-old male patient, who presented typical features of HLH in clinical examination, including hepatomegaly, low platelet counts, decreased NK cell activity, and hemophagocytosis, but was finally diagnosed with XLP1 by amplicon sequencing. A likely pathogenic nonsense mutation, NM_002351.4:c.300T>A, was identified by amplicon sequencing and confirmed by Sanger sequencing. X-linked recessive inheritance was identified based on a two-generation pedigree analysis. Gel electrophoresis of the RT-PCR product showed a truncated mRNA sequence of the *SH2D1A* gene in the patient.

The *SH2D1A* gene encodes the SH2 domain in SLAM-associated protein (SAP). To our knowledge, this domain consists of a phosphotyrosine binding pocket (pTyr-binding pocket), hydrophobic binding pocket, and a βG strand, which is highly important for SLAM binding [9]. Hence, mutations within the boundaries of the domain could directly implicate the SH2 domain in the pathogenesis of XLP [10]. However, the nonsense mutation we detected in this study is located around the βG strand (p.Tyr100X) and results in a loss of function of SAP.

XLP1 is a primary immunodeficiency caused by mutations in *SH2D1A*; another subtype of XLP is X-linked lymphoproliferative syndrome, type 2 (XLP2), for which the causative gene is *XIAP*, located on chromosome Xq25. Both of these two subtypes could be triggered by virus infection and present a strong resemblance to HLH. Detailed descriptions of XLP1 and XLP2 have been provided, including their clinical features, molecular genetics, and pathophysiology [11, 12]. However, the mutation spectrum and epidemiological features of this disease in China are unclear. Our results expand the spectrum of pathogenic mutations in *SH2D1A*, and may contribute to further epidemiological surveys of XLP.

Differential diagnosis of HLH is essential to ensure appropriate treatment. The phenotypes of XLP1 and HLH are highly similar, necessitating a rapid and accurate approach for differential diagnosis. Since the genetic factors of XLP and most subtypes of HLH are clear, amplicon sequencing could be an efficient and cost-saving method. In this case, all mutations in the target region were sequenced and analyzed in 24 h, with a cost of less than 100 dollars. Furthermore, our findings demonstrate that this technology can be used for the identification of novel mutations, in addition to known ones; thus, they have has implications for the improvement of genetic counseling and diagnosis for inherited diseases.

In conclusion, we used amplicon sequencing to identify a novel nonsense mutation in *SH2D1A* in a male patient with XLP1; the mutation was inherited from his mother, according to the X-linked recessive mode of inheritance. The RT-PCR results showed a truncated mRNA sequence in the patient due to the mutation, indicating a loss or partial loss of protein function. This finding also extends the spectrum of known XLP-related mutations in Chinese patients, and demonstrates the utility of amplicon sequencing for the identification of novel mutations and for differential diagnosis.

## Additional file

Additional file 1: **Table S1.** Information of primers for RT-PCR of *SH2D1A* gene. (DOCX 16 kb)

## Abbreviations

HLH: hemophagocytic lymphohistiocytosis
XLP1: X-linked lymphoproliferative syndrome, type 1
