# Supplementary material for: Identification of a novel nonsense mutation in SH2D1A in a patient with X-linked lymphoproliferative syndrome type 1: a case report
Source: BMC Med Genet. 2018 Apr 12;19:60. doi: 10.1186/s12881-018-0576-y (PMC5897942; doi:10.1186/s12881-018-0576-y)
Supplement: Supplementary file 1 — Table S1. Information of primers for RT-PCR of SH2D1A gene. (DOCX 16 kb) [file 12881_2018_576_MOESM1_ESM.docx]

**Table S1.** Information of primers for RT-PCR of *SH2D1A* gene.

| **Name** | **Sequence (5’-3’)** | **Template strand** | **Length** | **Start *** | **Stop** | **Product length with forward primer **** |
| --- | --- | --- | --- | --- | --- | --- |
| Forward | TTGCACAGTTCTCCTCCTCG | Plus | 20nt | 318 | 337 | - |
| Reverse 1 | TCAGGATCTTCTCTTATCCCTGT | Minus | 23nt | 726 | 704 | 409bp |
| Reverse 2 | TGCCTTGATCTGGCTTCTGA | Minus | 20nt | 641 | 622 | 324bp |

* Primers were designed using Primer-BLAST with a reference sequence from NCBI: NM_002351.4 (<https://www.ncbi.nlm.nih.gov/nuccore/295054105/>).

** Two reverse primers were matched with the forward primer respectively.
